# Proactive automatized multiple health risk behavior change intervention: reach and retention among general hospital patients

**DOI:** 10.1093/eurpub/ckaf035

**Published:** 2025-03-18

**Authors:** Marie Spielmann, Filipa Krolo-Wicovsky, Anika Tiede, Ulrich John, Jennis Freyer-Adam

**Affiliations:** Department of Prevention Research and Social Medicine, Institute for Community Medicine, University Medicine Greifswald, Greifswald, Germany; Institute for Medical Psychology, University Medicine Greifswald, Greifswald, Germany; German Center for Cardiovascular Research, Site Greifswald, Greifswald, Germany; Institute for Medical Psychology, University Medicine Greifswald, Greifswald, Germany; German Center for Cardiovascular Research, Site Greifswald, Greifswald, Germany; Department of Prevention Research and Social Medicine, Institute for Community Medicine, University Medicine Greifswald, Greifswald, Germany; Department of Prevention Research and Social Medicine, Institute for Community Medicine, University Medicine Greifswald, Greifswald, Germany; German Center for Cardiovascular Research, Site Greifswald, Greifswald, Germany

## Abstract

Although behavior change interventions are highly recommended in health care, their reach, a core dimension of the public health impact of interventions, is rarely evaluated. This study aimed to investigate whether an individualized, computer-based brief intervention to reduce co-occurring health risk behaviors (HRBs), namely tobacco smoking, at-risk alcohol use, insufficient physical activity, and unhealthy diet, can reach and retain a sufficiently large part of general hospital patients (>75%) and whether patients with high need, that is with more HRBs, low school education or current unemployment may be sufficiently reached and retained. Over 6 weeks in 2022, all 18–64-year-old patients admitted to 11 wards of five medical departments of a university hospital in Germany were asked to participate in a computer-based HRB screening and in a pre-post intervention study with three further assessments and individualized computer-generated feedback. To investigate associations between intervention reach and retention and patient characteristics, a logistic and a Poisson regression analysis were used. Screening reached 78.9% of all eligible patients (225/285). Of those eligible for the intervention study, 81.8% (175/214) participated in the intervention. Among these, 76.0% (133/175) participated at least once more after hospitalization. Patients’ lifestyle and socio-economic characteristics were not significantly associated with reach or retention, *P*s ≥ .467. Proactive computer-based multiple-HRB change interventions may reach and retain a sufficiently large part of general hospital patients, including those most in need. When proven efficacious and adequately implemented, this is a promising approach concerning public health impact in the reduction non-communicable diseases.

**Trial registration:**  ClinicalTrials.gov NCT05365269, 9 May 2022.

## Introduction

Tobacco smoking, alcohol at-risk drinking, unhealthy diet, and insufficient physical activity contribute to morbidity and mortality [1]. These four health risk behaviors (HRBs) are associated with mortality in a dose-response manner [2, 3]. More than half of the adult population and healthcare populations in Western countries show two or more of these four HRBs [4–6]. To prevent cardiovascular diseases, cancer, and other noncommunicable diseases, the development and implementation of cost-effective behavior change interventions in health care is recommended [7]. Assessing and addressing co-occurring HRBs is considered the future of preventive medicine [8]. From a public health perspective, two aspects should be in the focus of such interventions: public health impact and social equity [9, 10].

In order to have impact on public health, any interventions should reach a sufficiently large and representative proportion of the total target population [10]. And among those who participate e.g. in screening procedure or in the program, a sufficiently large and representative proportion should continue to participate (retention). This said, behavioral interventions should reach people who are most in need [10], e.g. those with particularly many HRBs. And to avoid further increase of social inequity in health, interventions should reach individuals with low level of school education or unemployed individuals at least as well as individuals with higher levels of school education or non-unemployed individuals [9]. This focus is crucial as these socioeconomic characteristics are (i) positively related to engaging in more HRBs [4, 5], which may contribute to the well-established higher morbidity and mortality [11–13]; and (ii) negatively related to participation in behavior change interventions or health promotion programs [14, 15]. Proactive intervention approaches that systematically and individually address and motivate each individual of the target population, can help to achieve higher participation and retention rates, also among most in need and hard to reach individuals [8, 16].

Hospitalization has been identified as a promising “window of opportunity” to reach people for behavior change interventions given easy access and the learnable moment [17, 18]. Despite rather low intention to change HRBs, 93% of hospital patients admitted for various reasons have been shown to approve systematic HRB screening and intervention for all patients at least somewhat; 58% reported high approval [19, 20]. And proactive recruitment has shown satisfactory reach for single-behavior change interventions with little selectivity in terms of socio-economic characteristics. For example, more than 80% of eligible patients with at-risk alcohol use were reached for brief alcohol interventions [16].

To further achieve public health impact, interventions must also be adequately adaptable, implementable and maintainable in the setting [10]. Digital multiple HRB change approaches may be helpful there, as they are cost- and time-saving and may reduce multiple HRBs [21, 22]. However, the proportion of the target population reached by web-based interventions and the representativity of the participating sample is often unknown; and drop out of participants over time is a common challenge [23, 24].

The aims of this study were to investigate: (i) whether a proactive HRB screening and computer-generated multiple-HRB change feedback would reach and retain a sufficiently high proportion (>75%) of all general hospital patients on various wards; and (ii) whether patients with lower school education, unemployment or with a particularly unhealthy lifestyle are at least as well reached for the multiple-HRB change intervention as their counterparts.

## Methods

### Sampling frame and screening

Data from the pre-post-intervention study “Proactive automatized lifestyle intervention for cancer prevention: Pilot-test” were used (PAL-Pilot; ClinicalTrials.gov: NCT05365269; 25). The ethics committee of the University Medicine Greifswald approved the study (BB 024/17; BB 024/17a).

Over 6 weeks between May 31 and July 5 in 2022, participants were recruited on 11 wards of five major medical departments of the university medicine hospital Greifswald in Germany: internal medicine A (gastroenterology, endocrinology, nephrology), internal medicine B (cardiology, angiology, pneumology), general and thorax surgery, trauma surgery, and otorhinolaryngology. On Tuesdays through Fridays, all patients aged 18–64 years and admitted the previous day were systematically and individually approached by a research assistant for HRB screening. Patients were individually approached and asked to fill in a survey on HRBs. Those who provided informed oral and electronic consent received a tablet computer to do so. Afterwards screening participants were individually asked to participate in the longitudinal pre-post intervention study. Excluded were patients cognitively or physically incapable to participate or terminally ill, patients with highly infectious diseases, patients discharged or transferred to outside the study area within the first 24 hours of their hospital stay, patients already recruited for the study during an earlier hospital stay, patients with insufficient language skills or employed at the conducting research institute or their hospitalized relatives. An additional exclusion criterion for participation in the intervention study was having neither telephone nor email as these contact details were required for subsequent study procedures. Those who provided written informed consent received the intervention.

### Intervention

As described in more detail elsewhere [25], the proactively recruited participants received individualized computer-generated feedback on their overall HRB profile and behavior change-enhancing feedback on up to two of their HRBs, i.e. on tobacco smoking, at-risk alcohol use, vegetable and fruit intake and/or insufficient physical activity. To avoid an overwhelming amount of feedback in the case of three or four present HRBs, two HRBs were selected by an algorithm based on a combination of scientific evidence and patients’ preferences. Automatized feedback was based on the participants’ assessment data, tailored to the current motivational stage of change [26], generated by computer-expert system software, and delivered by ordinary letter after assessments at baseline, month 1 and month 3. Online contact and feedback were not realized due to problems with information technology support during the pandemic. Participants who had provided email contact data only (*n* = 14) were emailed and asked to provide their telephone number.

### Measurements

#### Reach and retention

Two indicators of reach were measured, i.e. participation in the screening and participation in the intervention. Participation in screening was assessed by the proportion of screening participants among all eligible patients. Participation in the intervention was assessed by the proportion of intervention participants among all survey participants eligible for the pre-post-intervention study. Intervention retention was measured by the continuity of participation, i.e. the proportions of participants who continued to participate after discharge at months 1 and 3, and the frequency of participation (one, two, three times). Indicators of effort for proactivity were the average number of contact attempts (emails or telephone calls) required to deliver one month 1 intervention and one month 3 intervention.

#### Patient characteristics

Patient characteristics were assessed through self-report data at baseline. The questionnaire is described elsewhere [25].

Socio-economic characteristics included school education and unemployment status. School education was categorized into low/ medium/ high level of school, derived from German school qualifications corresponding to <10/10–11/>11 years of school, respectively. Unemployment status was assessed by two questions. Participants responding “no” on the first question “Do you have a job currently?” (yes, full-time/ yes, part-time 15–34 h/ yes, part-time <15 h/ no) were further asked which of eight response options applied (student/ unemployed <6 months/ unemployed between 6 months and 2 years/ unemployed > 2 years/ housewife or -husband/ military or voluntary service/ parental leave/ retired). Those reporting current unemployment were considered unemployed regardless of duration. All others were considered not unemployed.

Indicators of unhealthy lifestyle included the four HRBs and their sum score. Tobacco smoking was assessed by the self-reported number of cigarettes smoked per day in the past 4 weeks. Any current tobacco smoking, i.e. daily or occasionally, was considered as HRB smoking. Current alcohol use was determined using the 3-item Alcohol Use Disorder Identification Test-Consumption score [27]. For females and males, the HRB at-risk alcohol use was identified for scores ≥4 and ≥5, respectively. Current vegetable and fruit intake was assessed by the question “How many servings of vegetables and fruits do you eat per day on average?”. Twelve serving examples such as one medium-sized carrot or apple were provided. Less than five servings were considered as HRB insufficient vegetable and fruit intake [28]. Physical activity was assessed using the number of minutes of physical activity in a typical week adapted from the European Health Interview Survey-Physical Activity Questionnaire [29]. It measures physical activity by reported walking and cycling in everyday life activities and by reported sports activities. To determine physical effort adjusted minutes of physical activities, we used three additional items. They assessed whether participants breathed or sweated harder or whether their hearts beat faster. For walking and cycling, response categories were no/yes, often/yes, always; and reported minutes were multiplied by 0/0.5/1, respectively. For sports, response categories were a little stronger/very much stronger/differing from time to time; and reported minutes were multiplied by 1/2/1.5, respectively. Thus, the total number of minutes of physical activity corresponded to minutes of moderate physical activity. In line with recommendations of the World Health Organization [30], minutes of vigorous activity were doubled, light activity was not counted as physical activity, and reporting less than 150 min of moderate physical activity per week was considered as HRB insufficient physical activity. To represent overall lifestyle, the sum of co-occurring HRBs was calculated with a potential range of 0–4.

Other patient characteristics included self-reported indicators of health, motivation to change present HRBs, and socio-demographic variables such as age in years, sex (female/male), and family status (not living in partnership/living in partnership). As “indicators of health,” the presence of non-communicable diseases (yes/no) and self-rated health were assessed. Cancer diseases, chronic cardiovascular diseases, chronic respiratory diseases, and diabetes mellitus were recorded by four questions “Have you ever been diagnosed with [cancer-/chronic cardiovascular-/chronic respiratory disease/diabetes mellitus] by a doctor?” with six response categories (I don’t know/no, and there is no such suspicion/no, but there is a suspicion at the moment/yes, during my current hospital stay/yes, during the past year/yes, more than 1 year ago). Any “yes”-the response was coded as “disease present”. “Self-rated health” was assessed by asking “How would you rank your own health in general?” with five response categories (poor-excellent, 0–4). This item is a reliable and independent predictor of mortality [31]. When a certain HRB was present, “motivation to change” was assessed using staging algorithms based on the transtheoretical model of intentional behavior change [26]. As described in more detail elsewhere [25], the stages of change to reduce alcohol use and to increase physical activity were assessed by adapted four-item measures [16, 32]; and the stages of change for non-smoking and eating a minimum of five servings of vegetable and fruit per day by adapted 1-item measures [32, 33]. In general, participants reported no intention to change the respective HRB were allocated to “no intention to change.” Participants who considered to/thought they should or intended to start changing within the next 6 months were allocated to “ambivalent to change.” Participants seriously planning to change within one month or seriously attempting to change with the last attempt lasting were allocated to “ready to change.”

### Statistical analysis

To determine participation in the screening and in the intervention and continuity of participation, the number of cases (*N*) and proportions (%) among eligible patients, among eligible screening participants, and among intervention participants were determined, respectively. To identify whether a representative part of the screening participants entered the intervention program (yes/no), a multivariate logistic regression analysis with all predictors and adjusted for all other patient characteristics was performed. Motivation to change was not included, as depending on HRB occurrence, different stages of change were assessed for each participant. To provide information on intervention participation by the motivation to change, according to proportions of patients having no intention to, being ambivalent to or ready to change the respective HRBs are given. To analyze differences between participants receiving one, two, or three interventions, a multivariate Poisson regression was performed with all predictors, patient characteristics, and intervention modules received (smoking, alcohol, physical activity, diet). Cases with missing values on cigarettes per day (*n* = 1), non-communicable diseases (*n* = 8), self-rated health (*n* = 8), and received intervention module (smoking: *n*  = 1, diet: *n*  = 3) were excluded from the respective analyses list-wise. Stata version 14.2 was used.

Power calculation revealed that the sample size of *n* = 214 was sufficient to identify medium-sized effects of Cohen’s *d* = 0.5 (80% test power, α = 0.05, two-tailed) between two independent groups of different sizes (e.g. *n*_1_=175, *n*_2_=39).

## Results

### Participation in the screening and sample characteristics

**Figure 1.**
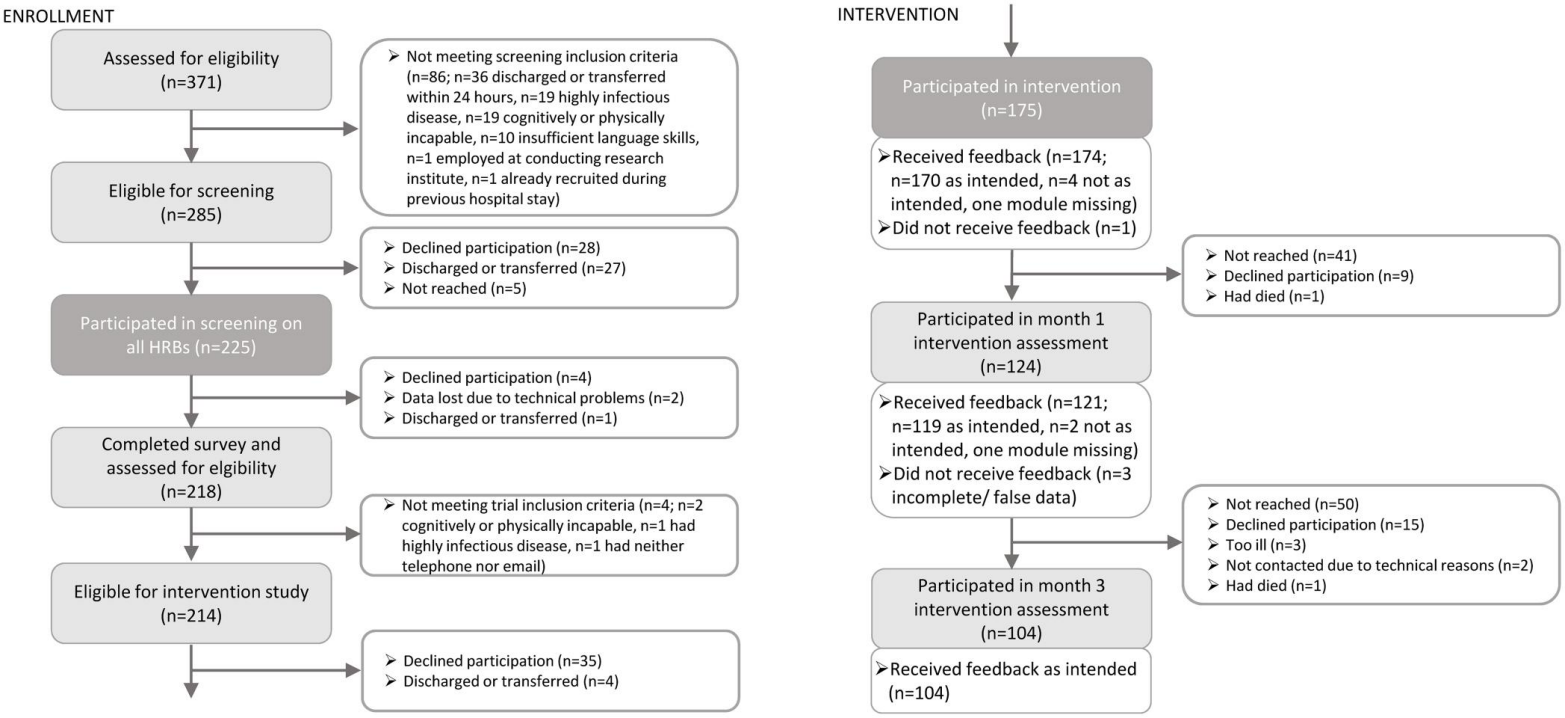
Flowchart according to CONSORT statement.

Of all eligible patients, 78.9% (225/285) participated in the screening and *n* = 218 completed the entire survey (Fig. 1). The mean age among all screening participants was 49.8 years (SD = 12.7), 56.0% (126/225) were male, and 24.4% (55/225) were married or living in a partnership. Sixteen percent (36/225) reported low, 63.6% (143/225) medium and 20.4% (46/225) high level of school; and 6.2% (14/225) were unemployed. The mean number of HRBs was 2.2 (SD = 0.9). Mean self-rated health was 2.7 (SD = 0.9); and 53.9% (117/217) had at least one non-communicable disease.

### Participation in the intervention

**Figure 2.**
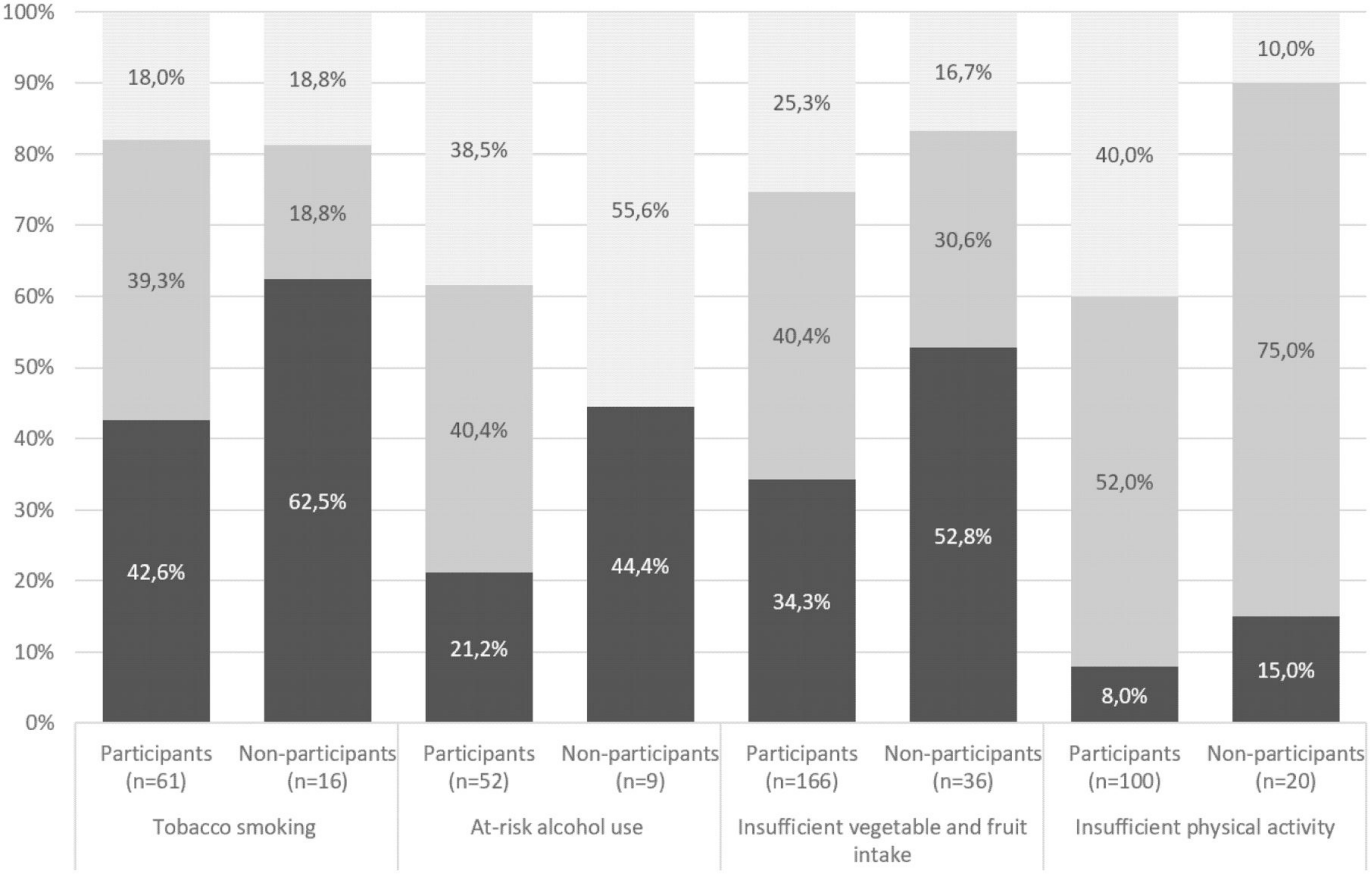
Proportions of patients not intending to change (black), ambivalent about change (gray), and ready to change (white) according to health risk behavior stratified by participation in intervention.

Of the screening participants eligible to participate in the trial, 81.8% (175/214) entered the intervention program and received their baseline intervention. Overall, 78.2% (61/78) of those with tobacco smoking, 85.3% (52/61) of those with at-risk alcohol use, 82.2% (166/202) of those with insufficient vegetable and fruit intake and 83.3% (100/120) of those with insufficient physical activity, participated in the intervention. Across all four HRBs, 60.0% (insufficient physical activity) to 82.0% (tobacco smoking) of those who participated in the intervention, were not yet ready to change their respective HRB (Fig. 2).

### Continuity of participation

Of the intervention participants, 76.0% (133/175) continued to participate in the intervention after hospitalization. That is, 70.9% (124/175) participated in month 1 and 59.4% (104/175) in month 3 (Fig. 1). Overall, 24.0% (42/175) participated at baseline only, 21.7% (38/175) in two, and 54.3% (95/175) in all three interventions.

In order to deliver the 1-month intervention to 124 participants, a total of 794 contact attempts were realized by research assistants, resulting in 6.4 contact attempts per delivered intervention. For the 3-month intervention, a total of 1008 contact attempts were realized for 104 delivered interventions, resulting in 9.7 contact attempts per delivered intervention. In total, the delivery of all 1-month and 3-month interventions required an average of 7.9 contact attempts per delivered intervention.

### Subgroup differences in intervention participation and its continuity

Both multivariate regression analyses revealed that neither socio-economic characteristics nor lifestyle variables were significantly associated with intervention participation (*P*s ≥ .467_vegetable/fruit_) and continuity of participation (*P*s ≥ .522_HRB number_, Table 1). Also, none of the socio-demographic (age, sex, partnership) and health-related (non-communicable disease, self-rated health) covariates were significantly related to initial intervention participation (*P*s ≥ .168_age_) and continuity of participation (*P*s ≥ .300_age_). Intervention module received was also not related to continuity of participation (*P*s ≥ .738_alcohol_).

**Table 1.** Sample characteristics stratified by intervention participation and frequency of participation

		Intervention participation (*n* = 214)				Frequency of participation (*n* = 175)			
		No (*n* = 39)		Yes (*n* = 175)		Once (*n* = 42)		2–3 times (*n* = 133)	
Socio-economics (*n*, %)									
Years of school	<10 years	7	17.95	26	14.86	8	19.05	18	13.53
	10–11 years	24	61.54	116	66.29	25	59.52	91	68.42
	>11 years	8	20.51	33	18.86	9	21.43	24	18.05
Unemployment	Not unemployed	36	92.31	165	94.29	39	92.86	126	94.74
	Unemployed	3	7.69	10	5.71	3	7.14	7	5.26
Health risk behaviors (M, SD)									
Cigarettes per day		4.73	7.57	4.77	8.43	7.35	9.44	3.96	7.95
Alcohol use disorder identification test-consumption		2.51	2.30	2.73	2.55	3.62	2.96	2.44	2.34
Servings of vegetables and fruit per day		2.13	1.79	2.25	2.08	2.00	2.24	2.33	2.03
Minutes physically active per week		273.08	359.88	253.27	401.16	208.39	282.76	267.44	431.8
Total number of health risk behaviors		2.08	0.90	2.17	0.91	2.55	0.92	2.05	0.88
Other patient characteristics									
Non-communicable disease (*n*, %)	Yes	21	53.85	95	54.60	24	58.54	71	53.38
	No	18	46.15	79	45.40	17	41.46	62	46.62
Self-rated health (M, SD)		2.62	0.94	2.68	0.86	2.68	0.69	2.68	0.91
Age (M, SD)		51.44	12.26	48.89	12.88	46.02	12.99	49.80	12.76
Sex (*n*, %)	Female	18	46.15	78	44.57	15	35.71	63	47.37
	Male	21	53.85	97	55.43	27	64.29	70	52.63
Living in partnership (*n*, %)	No	9	23.08	43	24.57	14	33.33	29	21.80
	Yes	30	76.92	132	75.43	28	66.67	104	78.20
Received intervention module (*n*, %)	Smoking	–	–	60	34.29	21	50.00	39	29.32
	Alcohol	–	–	33	18.86	10	23.81	23	17.29
	Diet	–	–	130	74.29	27	64.29	103	77.44
	Physical activity	–	–	80	45.71	21	50.00	59	44.36

*Notes:* M, mean; SD, standard deviation; *n*, number of cases.

## Discussion

The three key findings of this study are: (i) The proactive and computer-based behavior change and prevention approach reached 79% of all general hospital patients for screening and 82% of the screening participants to participate in the intervention (study), of whom 76% continued to participate after hospitalization. (ii) With 60% and more, the majority of successfully reached patients were not yet intending to change or were ambivalent to change their HRBs. (iii) Reach and retention of patients with more HRBs, low level of school education or unemployment were as high as reach and retention among patients with fewer HRBs, higher education, and non-unemployed patients.

Our findings confirm encouraging findings from previous proactive screening and brief interventions on single HRBs in healthcare settings: once patients are successfully reached for screening, the proportion of those reached for and retained in the intervention program is high [16, 34]. However, although high, the screening reach of 78% was lower compared to 92% found in the same hospital 10 years earlier [16]. This discrepancy may largely be explained by recruitment taking place during the COVID-19 pandemic, which posed significant challenges on research concerning non-COVID-19-related studies [35]. For example, patients were discharged or transferred more quickly compared to the former study, resulting in more patients being missed for screening (9% vs. 4% of eligibles, 16). However, intervention reach and retention were rather comparable to findings from proactive interventions in the general hospital setting ten years ago and in other settings [16, 34, 36].

As intended, and in line with previous high-effort proactive intervention studies [16, 34], and with 60% and more of the intervention participants not yet ready to change their HRBs [20], this study’s proactive approach succeeded in reaching the crucial majority of the target population, namely also those patients who are rather unlikely to actively seek advice without being proactively approached; and who rather require different treatment than patients already committed to change [26]. These findings further underline that approaches involving a combination of both, high-effort proactivity and motivation-enhancing interventions tailored to the individuals’ current motivational stage represent the future of preventive medicine [8]; and according to trials have been shown to address these challenges successfully [37].

In combination with previous study results, these findings highlight high-effort proactivity as a core ingredient of behavioral interventions to not further increase social inequity in health [9, 38]. In line with a similar high-effort proactive intervention approach in various healthcare settings [39], and in contrast to previous lower-effort proactive approaches [14, 15], intervention reach and retention were independent of the number of co-occurring HRBs and socio-economic characteristics. For example, 79% of the patients with no or one HRB, 83% of those with all four HRBs, 74% of the patients with low level of school, and 81%/73% of those with medium/high level of school participated in the intervention.

Considering that the majority of general hospital patients (66%) report multiple HRBs [6, 39], the general hospital seems to be a promising location to implement behavior change interventions for the prevention of non-communicable diseases. Patients reached through interventions in the general hospital might be older and have a lower general health status [39]. However, they may be approached in an easy and time-saving way during routine care. In addition, increased motivation to change HRBs as found for alcohol use or tobacco smoking during hospitalization [18, 40], and patients’ openness toward systematic HRB screening and intervention during hospitalization [19] open up a window of opportunity for brief interventions targeting multiple HRBs [18]. Given the high reach and retention of hospital patients regardless of motivation to change, number of HRBs, and socioeconomic characteristics, proactive multiple-HRB change interventions have the potential to support the prevention and treatment of cancer and other non-communicable diseases in healthcare settings.

A few strengths and limitations of this study are to be noted. Strengths include firstly, that the intervention tested was designed to reach all patients, especially those most in need by applying a proactive recruitment strategy to reduce selection bias. Secondly, patients from five medical departments were included, allowing conclusions beyond certain patient groups. Limitations included firstly, that all assessments were based on self-report which may lead to providing socially desirable responses. However, self-report is the foundation of behavior change interventions and of individualized motivation-enhancing feedback required for a large part of not-yet-motivated patients in particular. To reduce bias, we used validated measures whenever possible. Secondly, given that 22% of the eligible patients did not participate in the initial survey, and as their characteristics are unknown; we cannot rule out that particularly hard-to-reach subgroups were not reached for screening. This may particularly apply to one-half of the non-participants who actively declined participation and who represented 11% of all eligible patients. The other half was not reached due to limitations rather related to the recruitment being implemented as part of a research study, e.g. study staff found patients to be discharged or referred to other wards or was unable to reach patients within pre-defined three working days. These limitations may not exist once proactive HRB screening and brief intervention are implemented into routine hospital care, e.g. provided by additional prevention staff. Thirdly, some small effects may not have been detected due to insufficient sample size. Fourthly, as discussed above, the comparability of the results is limited due to recruitment taking place during the COVID-19 pandemic.

To conclude, proactively delivered computer-based, theory-driven, and motivation-enhancing multiple-HRB change interventions may be considered a promising tool in terms of target reach, a core dimension of the public health impact of behavioral interventions. They may reach and retain large and representative proportions of hospitalized patients for prevention and health promotion purposes. The findings support that proactivity may help to reduce severe selection bias in terms of social inequity in health [16] by reaching subgroups of individuals who are most in need of behavioral intervention [8] and often hard to reach [15]. If proven efficacious and adequately implemented, proactive and theory-driven computer-based multiple-HRB change interventions may have the potential to achieve impact on public health in terms of helping to prevent and treat non-communicable diseases in health care settings.

## Data Availability

The datasets generated and analyzed during the current study are not publicly available due to the German data protection law but are available from the principal investigator of the study, Prof. Dr Jennis Freyer-Adam on reasonable request that complies with the study purpose and the participants informed consent. Key points A proactive, computer-based multiple health risk behavior change approach may reach 82% of proactively approached general hospital patients; and retain 76% of them after hospitalization. Patients with more health risk behaviors, low school level, or unemployment were as well reached as patients with fewer health risk behaviors, higher level of school, or non-unemployed. With the majority of intervention participants not yet intending or ambivalent to change their health risk behaviors, proactivity succeeded in reaching patients who would not yet seek advice by themselves.
